# Foetal haemoglobin-blood cells (F-cells) as a feature of embryonic tumours (blastomas)

**DOI:** 10.1038/sj.bjc.6603867

**Published:** 2007-06-26

**Authors:** M Wolk, J E Martin, M Nowicki

**Affiliations:** 1 Department of Histopathology, Royal London Hospital, Centre for pathology, Institute of Cell and Molecular Sciences, Queen Mary School of Medicine and Dentistry, Pathology and Pharmacy Building, The Royal London Hospital, 80 Newark Street, Whitechapel, London E1 2ES, UK; 2 Department of Histology and Embryology, Poznan University of Medical Sciences, Swiecickiego 6, Poznan 60-781, Poland

**Keywords:** foetal haemoglobin, tumour marker, embryonic tumours, DNA hypomethylation, childhood cancer

## Abstract

Tumour markers are important in the diagnosis and monitoring of many tumours. This study tested the hypothesis that an oncofoetal protein, foetal haemoglobin (HbF) is a potential tumour marker in embryonic tumours, useful for management. An immunohistochemical investigation of HbF blood cell (Fc) distribution was carried out in tumours and in bone marrow samples from 83 children and 13 adults with various embryonic tumours (blastomas), and in bone marrow samples of 24 leukaemia patients. In the three, main blastoma types, nephroblastoma (Wilms' tumour), neuroblastoma and retinoblastoma, where all the patients, except two, were children, around 80% of the tumour samples had Fc within proliferating blood vessels and spaces between tumour cells. In parallel, clusters of Fc, mostly F-erythroblasts (Feb), were distributed in the bone marrow of some of those patients and in the bone marrow of 79% of the leukaemia patients. Foetal haemoglobin, as well as being a potential prognostic cancer marker, is a potential indicator of DNA hypomethylation implicated in the development of these tumours, as well as in others previously noted for the presence of HbF.

Elevated concentrations of foetal haemoglobin (HbF) are common in the predominant kinds of solid tumours as well as in haematological malignancies (Wolk *et al*, 1991, 1999). In preliminary follow-up studies conducted on childhood leukaemia (Rautonen and Siimes, 1990), in myelodysplastic syndrome (Reinhardt *et al*, 1998) and in non-Hodgkin's lymphoma (Wolk and Newlands, 2004), HbF has been evaluated as a valid prognostic parameter, which should be considered in the management of the disease. There is evidence that HbF inducing growth factors are raised in cancer patients, including stem cell growth factor (CSF) and interleukin-3 in the serum of colorectal cancer patients (Mroczko *et al*, 2003), and a growth factor found in the bone marrow of patients with myelodysplastic syndrome (Choi *et al*, 2002). However, the mechanisms for renewal of globin *γ*-chain gene expression in cancer patients are yet not clear. Foetal haemoglobin in most cases is not a product of the tumour cells. Foetal haemoglobin expression by the blood cells surrounding the tumour appears to be induced under the conditions of malignancy. Defining these conditions might contribute to the understanding of carcinogenesis at its basic levels. In this respect, we note that DNA hypomethylation (DNhpom) at CpG dinucleotides (cytosine nucleotide found adjacent to guanine nucleotide) is an example of an epigenetic process implicated in the promotion of carcinogenesis (Ehrlich, 2005), as well as in the activation of globin *γ*-chain gene expression (Atweh *et al*, 2003; Lavelle, 2004). For example, DNhpom was proved experimentally to promote hepatocellular carcinogenesis (Yamada *et al*, 2005) and T-cell lymphoma (Gaudet *et al*, 2003), and to correlate with recurrence in hepatocellular carcinoma (Itano *et al*, 2002). In parallel, a DNA hypomethylating cytidine analogue drug given to lung cancer patients (Carr *et al*, 1987) and to patients with sickle cell disease (Saunthararajah *et al*, 2004) increased their HbF concentrations. In the sickle cell patients, HbF rose to 22% (normal level is<1%). Hence, DNhpom agents might promote neoplasia in prospective tumour cells simultaneously with reactivation of HbF gene expression in erythrocytes precursors, residing inside the tumour tissues or in haematopoietic tissues of the same organism. This hypothesis is not impaired by the evidence for inactivation of tumour suppressing genes by DNA hypermethylation, since hypermethylation is specific for unique genomic sites while at the same time global DNA is hypomethylated (Ehrlich *et al*, 2002; Brothman *et al*, 2005; Ehrlich, 2005). By that ambiguity, the use of DNA demethylating agents as therapeutic anticancer regiment might contribute to carcinogenesis (Ehrlich, 2005). By establishing the association between carcinogenesis and HbF via DNhpom, detection of HbF might contribute to therapeutic strategies, and to the alerting of hypomethylating agents in the environment, suspected as carcinogenic hazards. The first indication for a possible link between DNhpom in tumour tissue (in the tissues of colorectal adenocarcinoma) and DNhpom of the *γ*-globin gene came by demonstrating hybridization between the two sources of DNA (Feinberg and Vogelstein, 1983). Since then, DNhpom has been found in a large variety of tumour tissues, including leukaemia (Yoshida *et al*, 2004), prostate (Brothman *et al*, 2005), breast (Van den Eyden *et al*, 2005), urinary bladder (Nakagawa *et al*, 2005) and others, most of them known for elevated HbF. Another group of solid tumours notable for DNhpom, is embryonic tumours (blastomas), which are most common in children.

The aim of this study was to investigate the presence of F-cells in blastoma-related tumours by the use of immunohistochemistry. In this way, conditions inducing HbF expression can be related locally to the tumour tissue, rather than to the whole organism, as in our study of colorectal tumours, where we detected local development of F-cells (Wolk *et al*, 2006).

Our study included Wilms' tumour (nephroblastoma), neuroblastoma, retinoblastoma, childhood leukaemia, rhabdomyosarcoma and others, all of which originate from primordial embryonic cells, including the embryonic neural crest as the presumed source of neuroblastoma, Ewing's sarcoma, medulloblastoma and rhabdomyosarcoma. All have similar morphology, with uniform, primitive embryonic, blastoma cells. Some of them are reported to share the same oncogenes, as WT1 in Wilms' tumour (Maiti *et al*, 2000), in retinoblastoma (Wagner *et al*, 2002) and in acute myeloid leukaemia (King-Underwood *et al*, 1996), and RB1 in retinoblastoma (Sanchez *et al*, 2000), neuroblastoma (Markaki *et al*, 2001), childhood leukaemia (Markaki *et al*, 2001) and Ewing's sarcoma (Cope *et al*, 2001). We evaluated HbF as a cancer marker in those diseases by examining the histological distribution of F-cells in the tumours and bone marrow of those patients, noting their concentrations and locations.

## MATERIALS AND METHODS

### Participants

The study was conducted in two institutions: (1) Pathology Group, Institute of Cell and Molecular Sciences, The Royal London Hospital, England and (2) Department of Histology and Embryology, Poznan University of Medical Sciences, Poland. The programme of research including studies on archival and stored materials was approved by The East London and City Health Authority Research Ethics Committee and by the Poznan University of Medical Sciences, Department of Paediatric Oncology, Haematology and Transplantology, Ethics Committee.

### Immunohistochemical staining

We used the peroxidase avidin–biotin method with an affinity purified anti-HbF (Wolk *et al*, 2004). All the reagents used, as well as the procedures of immunostaining confirmed by control staining, were as described in our previous work (Wolk *et al*, 2004). The intensity of staining was indicated by five grades from (+) to (+++++).

## RESULTS

### General

The study included examination of histological tumour and bone marrow specimens of the following diseases: nephroblastoma (*n*=24), leukaemia (*n*=24), retinoblastoma (*n*=18), neuroblastoma (*n*=17), rhabdomyosarcoma (*n*=14), medulloblastoma (*n*=11), Ewing's sarcoma (*n*=2), hepatoblastoma, neurofibroma and glioblastoma (*n*=1 for each). All these patients, except those with leukaemia and medulloblastoma, were predominantly children.

**Figure 1:**
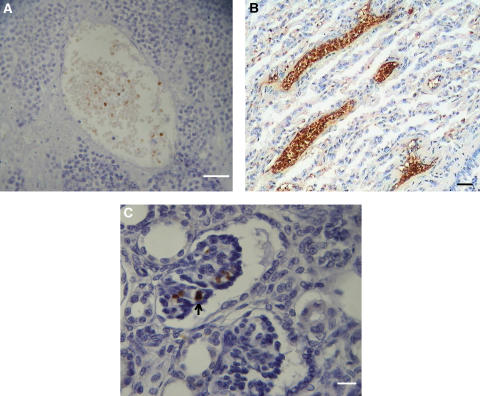
Nephroblastoma. (**A**) Large blood vessel in tumour tissue, containing 20% Fer. Calibration bar, 50 *μ*m. (**B**) Tubular differentiating area with proliferating blood vessels full of Fc. Calibration bar, 50 *μ*m. (**C**) Fc within glomeruloid body. Arrow indicates one Feb. Calibration bar, 25 *μ*m.

**Figure 2:**
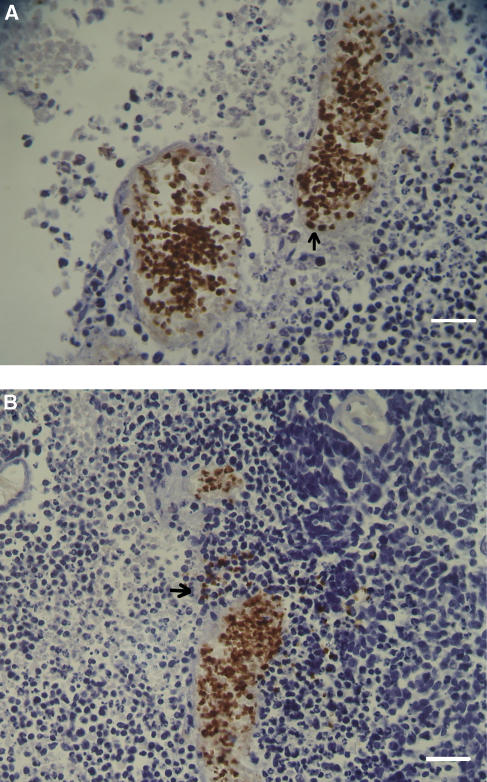
Retinoblastoma. (**A**) Proliferating blood vessels full of Fc. Thirty percent are (nucleated) Feb, two of which are indicated by arrow. Calibration bar, 50 *μ*m. (**B**) A condensation of extravascular Fc (indicated by arrow) between the two blood vessels of Fc. Calibration bar, 50 *μ*m.

**Figure 3:**
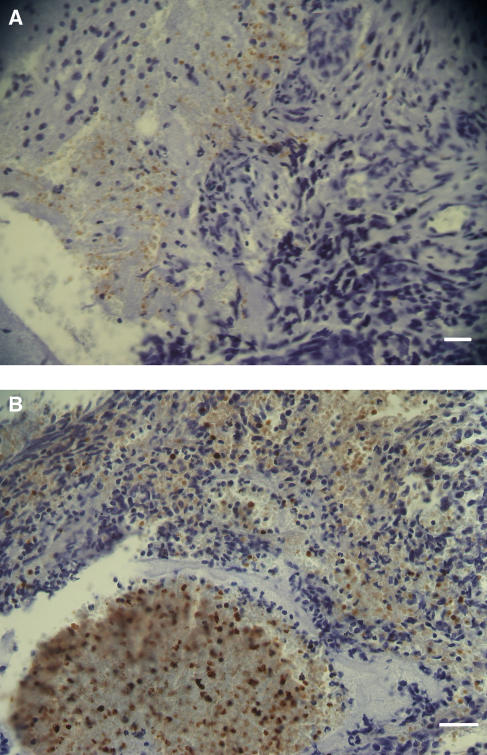
(**A**) Infiltrating Fc (orange immunostained) in neuroblastoma of the brain. To the left is a brain tissue free of Fc. Calibration bar, 50 *μ*m. (**B**) Neurofibroma; an example of congested Fc in a necrotic area (bellow) and Fc infiltrated into tumour tissue (above). Calibration bar, 50 *μ*m.

**Figure 4:**
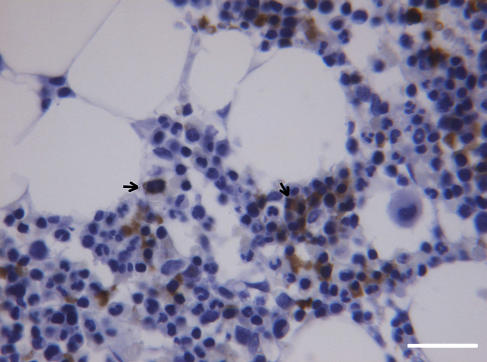
Rhabdomyosarcoma. Bone marrow with a network forming clusters of Fer and Feb. Arrows indicate two Feb in mitosis. Calibration bar, 50 *μ*m.

**Figure 5:**
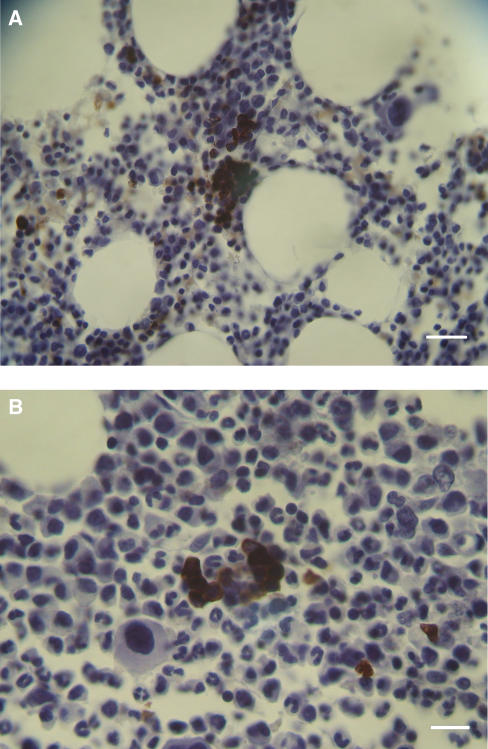
Leukemia. (**A**) Bone marrow from hairy cell leukaemia with a large concentration of Feb and other dispersed. Feb and Fer. Calibration bar, 50 *μ*m. (**B**) A cluster of Feb in a bone marrow from CML. Calibration bar, 25 *μ*m.

Foetal haemoglobin blood cells (Fc) comprised mature erythrocytes (Fer) and nucleated erythroblasts (Feb), which could sometimes be binucleated or in mitosis. Foetal haemoglobin blood cells were identified and were distributed in blood vessels or free in tumour tissues. In the blood vessels their concentrations were 0–100%. They could be distributed either randomly in the blood vessels, in most of the cases not exceeding 50% of the total blood cells (Figure 1A), or at 100% inside proliferating, closely aligned blood vessels (Figures 1B and 2A). In the following tables, cases classified as (1) ‘low-percent Fer’ (Fer+), were those with 0–30% Fc per one blood vessel, in which usually no more than 20% of the vessels contained Fc; (2)‘high-percent Fer’ (Fer++) were those with >30–100% Fc per one blood vessel, where usually 30–100% of the blood vessels contained Fc. Close to the proliferating blood vessels, there were packed free extravascular clusters of Fc, including many Feb, as in proliferation centres (Figure 2B). Usually Fc were also observed infiltrated among the tumour cells and congested in large areas of necrosis or haemorrhage (Figures 3A and B). In bone marrow, F-cells were observed in dense clusters of 3–50 cells distributed through the whole section (Figures 4 and 5).

The scarce cases treated by chemotherapy prior to our examination were noted in the tables of results.

### Nephroblastoma

Table 1 summarizes the immunohistochemical distribution of Fc in 24 patients with tumours. Foetal haemoglobin blood cells were detected in 19 (79%) of these patients, all of them children aged 1–14 years (Table 1, numbers 1–9, 14–23). Control specimens included one normal kidney (Table 1, number 22), seven normal kidney tissues adjacent to a removed tumour (Table 1, numbers 10–13, 15, 16, 24) and three kidney tissues of patients with glomerulonephritis (Table 1, numbers 31–33). No Fc were observed in these controls, except one case (Table 1, number 10). Ten patients (Table 1, numbers 1–10) were followed up until the present time. However, no correlation could be found between the last clinical stage reported and the Fc distribution. All the tumours were of three-phase nephroblastoma, including all the three cellular components, namely: blastoma cells, mesenchyme and epithelial cells forming tubular elements. Foetal haemoglobin blood was found in each of these components, including tubular structures (Table 1, numbers 9, 10, 23) and glomeroid bodies (Table 1, number 7, 9; Figure 1C), as well as in the blood vessels (Table 1, numbers 1–8, 10, 15, 16, 17, 19, 20, 21; Figure 1A) Congested Fc were very common in large necrotic and haemorrhagic areas, or between tumour cells. Evidence was also found for proliferating blood vessels full of Fc (Table 1, numbers 2, 3, 8; Figure 1B).

### Neuroblastoma

The results are summarized in Table 2. Tumour specimens were examined in 16 children, 2/12–14 years old and one adult. Twelve of them (75%) were Fc positive. Foetal haemoglobin blood cells, mainly Fer, were infiltrated among the tumour cells (as in Figures 3A and B) and randomly distributed inside blood vessels. No F-cells were found in ganglioneuroma (Table 2, number 5) or in brain tissue adjacent to the tumour tissue (Table 2, number 6; Figure 3A). No correlation could be found between the clinical condition and the distribution of Fc. In the bone marrow with metastases, high concentrations of Feb were observed in one patient (Table 2, number 14), while three other bone marrow samples were negative.

### Retinoblastoma

The results are summarized in Table 3. Here again all patients were very young infants, most of them 3 years and younger. Eleven (78.6%) of the 14 tumour specimens were Fc positive. Non-metastatic bone marrow specimens from three out of four other patients were strongly positive for Fc, mostly Feb. The expression of foetal haemoglobin and the percentages of Feb were very prominent, exceeding all other tumour types examined here.

Some of the Feb were binucleated or in mitosis. We noted here the high rate of proliferating Fc in regenerating blood vessels and in free cell clusters (Table 3, numbers 1–4; Figures 2A and B)

### Rhabdomyosarcoma

The results are summarized in Table 4. Eight (89%) of the nine tumour specimens were Fc positive. Tumour specimens were from seven children, 1–8 years old, and two adults. In tumour blood vessels, the F-cells were randomly distributed. Free Fc were distributed in tumour tissues as in the groups above. In bone marrow specimens from two of five patients, there were clusters of Fc, and high-rate Feb mitosis was noted in one of them (Figure 4).

### Medulloblastoma

All specimens were from tumour in the cerebellum. As described in Table 5, five patients were children 5–15 years old and six other were young adults. Six of the 11 patients were Fc positive, with the characteristic distribution as above.

### Leukaemia

A list of Fc positive and negative cases is shown in Table 6. Bone marrow specimens were examined from 24 patients; 19 (79%) of them were Fc positive. Seven patients were boys and girls aged 2–14 years. The remaining 17 adult patients were aged between 31 and 83 years. Our initial aim was to examine childhood leukaemia because it is considered as leukoblastoma (van den Berg, 2002). However, our results indicated that the incidence and magnitude of Fc distribution were the same for children and adults. There was also no difference with regards to the type of leukaemia. Foetal haemoglobin blood cells were distributed through the whole sections of the bone marrow as cluster of 3 to 50 cells, most of them Feb. All of them were strongly stained (+++++), as shown in Figures 5A and B.

#### Ewing's sarcoma

One tumour specimen, from the temporal bone of a 60-year-old man, had Fc distributed at 10–50% in part of the blood vessels and in necrotic areas. About 1/4 of these Fc were Feb. Another sacral tumour from a 2-year-old boy had a few fine blood vessels with 100% Fer.

#### Neurofibroma

In a tumour specimen from a cyst in the leg of 1-year-old boy, we observed Fc cells infiltrating among tumour cells and in adjacent necrotic areas, where some were Feb, as shown in Figure 3B.

#### Glioblastoma

Tumour specimen from the brain of a 52-year-old woman consisted of blastoma-like cells infiltrated by Fer. The adjacent brain tissue was free of Fc.

#### Hepatoblastoma

In a bone marrow specimen from a 2-year-old girl, we observed Fc, most of them Feb, distributed as foci and as single cells.

## DISCUSSION

As described in the results, F-cells (Fc) were distributed in various patterns inside the embryonic tumour tissues. Foetal haemoglobin blood cells probably originate in the bone marrow and, delivered by circulation, were found randomly at 0–50% in the main blood vessels (Figure 1A). However, Fc including Feb comprised up to 100% of the contents of proliferating blood vessels and as packed free clusters (Figures 2A and B) could have originated in the tumour tissue. It seems therefore that there are two sources of Fc. One is in the bone marrow leading to elevated whole-blood HbF concentration, and the other is in the tumour tissue leading to elevated plasma HbF concentration. A support for this assumption is found in our previous works showing that plasma HbF and whole-blood HbF are two independent indicators in many kinds of cancer (Wolk *et al*, 1999). One of these cancers was non-Hodgkin's lymphoma, in the patients of which we have immunohistochemically detected Fc in lymph nodes and in bone marrow (Wolk *et al*, 2004), which respectively might have been the sources for (1) plasma HbF and (2) whole-blood HbF. We have found similar reciprocation (same references) in teratoma. However, these assumed correlations between the immunohistochemical and the serological findings have yet to be confirmed by concomitantly performing both essays. In our four main tumour groups comprising nephroblastoma, neuroblastoma, retinoblastoma and rhabdomyosarcoma, within the 60 cases of childhood embryonic tumours, we found prominent HbF expression in up to 80% of patients. This is the highest percentage of HbF ever detected immunohistochemically in cancer, which makes it an important feature of childhood embryonic tumour. Foetal haemoglobin measurements should therefore be considered in the management of those tumours, as the concentrations might change with regression or progression of the tumours. Foetal haemoglobin blood cells, most of them being Feb, were detected predominantly in the bone marrow of patients with leukaemia, but also in neuroblastoma, rhabdomyosarcoma, hapatoblastoma and especially in retinoblastoma. Since bone marrow is the likely source for circulating Fc, its Fc-immunohistochemical evaluation could be an early sign for the onset of those diseases, as for example in childhood leukaemia where HbF has already been proved as a prognostic marker (Rautonen and Siimes, 1990). In those cases the examination of bone marrow is advantageous over other tissue examinations, because sampling bone marrow aspirates is easier than collecting biopsies or resected specimens. In our introduction, we noted the possible association between the HbF gene reactivation and DNhpom implicated in oncogenesis. A support for this association is found in the recent literature noting the importance of DNhpom in embryonic tumours, found here to contain high concentration of F-cells, including nephroblastoma (Mares *et al*, 2001) and neuroblastoma (Banelli *et al*, 2005). However, DNhpom is not always involved in the mechanisms of reactivation HbF gene re-expression in cancer. There are yet other hypothetical pathways not involving DNhpom. For example the involvement of hydroxyurea – an important drug for inducing HbF biosynthesis (Atweh *et al*, 2003), which like DNA demethylation agents may promotes carcinogenesis (De Benedittis *et al*, 2004).

Another aspect of the abundance of F-cells inside these tumours is related to their embryonic characteristics. Some of these tumours mimic normal embryonic development, such as three-phase nephroblastoma, containing mesenchyme, tubular and glomerular elements, or neuroblastoma cells, which may differentiate to ganglion cells or neural cells. It is therefore possible that these tumours are programmed for autonomous foetal haematopoiesis by F-cell precursors. This hypothesis is supported by the finding of HbF erythroblasts (Feb), some of them in mitosis, in all of these embryonic tumours, most prominently in retinoblastoma and nephroblastoma. Another support for Fc development in tumour tissue comes from the finding of Fc, including Feb, in proliferating blood vessels. These Fc were found only in the tumour and not in other adjacent tissues like normal kidney tissue adjacent to nephroblastoma (Table 1), or brain tissue adjacent to glioblastoma, and neuroblastoma (Table 2, number 6; Figure 3A).

It seems thus that local regeneration rather than transportation through the general circulation is the source of Fc among tumour cells, and in proliferating blood vessels inside the tumours.

## Figures and Tables

**Table 1:** The distribution of F-cells immunohistochemically detected in nephroblastoma

				**F-cells features**						
**No.**	**Gender**	**Age (years)**	**Clinical parameters**	**BV**	**PBV**	**TC**	**GL**	**TB**	**NK**	**Staining grade**
1	Female	6	Stage IV, pulmonary metastases, died	Fer++	(−)	Fer	(−)	(−)		(+++)
2	Female	7	Stage III, died	Fer++	Fer	Fer				(+++++)
3	Female	8	Stage III, died		Fer	No Fc				(+++)
4	Male	5	Stage IV, pulmonary metastases, remission, relapse, remission	Fer+	(−)	No Fc	(−)	(−)		(+)
5	Male	4	Stage III, remission	Fer++	(−)	Fer,Feb	(−)	(−)		(+++++)
6	Male	8	Stage III, remission	Fer++	(−)					(++)
7	Female	13	Stage III, remission	Fer,Feb+		Fer,Feb	Fer,Feb			(+++++)
8	Female	11	Stage III ,remission	Fer++	Fer	No Fc				(+++++)
9	Male	3	Stage III, relapse and remission	(−)	(−)	No Fc	Fer	Fer		(++++)
10	Male	2	Stage III, remission, adjacent NK	Fer+	(−)	No Fc	(−)	Fer		<(+)
11–13	Females	4, 5, 9	Stage II, remission, adjacent NK	No Fc						(−)
14	Male	2	Metastatic stage IV, lung with metastases. Chemother(+)	(−)	(−)	Fer				(++++)
15	Male	6	Stage III+adjacent NK	Fer+	(−)	No Fc			No Fc	(+++)
16	Female	1	Stage III+adjacent NK	Fer+	(−)	No Fc			No Fc	(+++)
17	Male	1	Stage II	Fer+		Feb	(−)	(−)		(+++++)
18	Male	7	Stage II	(−)	(−)	Fer	(−)	(−)		(+++)
19	Female	2	Stage I	Fer+	(−)	Feb	(−)	(−)		
20	Female	3	Stage I	Fer+	(−)	Fer	(−)	(−)		(+++)
21	Male	1	Stage I	Fer+	(−)	Fer	(−)	(−)		(+++)
22	Female	4	Stage I+counterpart NK. Chemoth(+)	(−)	(−)	Fer	(−)	(−)	No Fc	(+++)
23	Male	14/12	Stage I	(−)	(−)	No Fc	(−)	Fer		(+++)
24	Female	1	Stage I adjacent NK. Chemoth(+)	No Fc						(−)
25–29	3 female 2 male	2, 3, 5 17, 22	Stage I (4), stage II (1)	No Fc						(−)
30–32	1 female 2 male	4, 7, 9	Kidney of glomerulonephritis	No Fc						(−)

Abbreviations: (−), tissue not present in the section; BV, normal blood vessels; Fc, F-cells; Feb, F-erythroblasts; Fer, F-erythrocytes; Fer+, low percent of FER; Fer++, high percent of Fer; GL, glomeruloid bodies; NK, normal kidney; PBV, proliferating blood vessels; TB, tubular structures; TC, undifferentiated tumour cells.

**Table 2:** The distribution of F-cells immunohistochemically detected in neuroblastoma

				**F-cells features (1) Checked in tumour tissues**				
**No**	**Gender**	**Age (years)**	**Clinical parameters**	**BV**	**NB**	**GN**	**BR**	**Staining grade**
1	Female	14	Stage IV adrenal NB with mets to bone marrow, died	Fer++	No Fc	(−)	(−)	(+ +)
2	Male	4	Stage IV, adrenal NB with mets to bone marrow, died	(−)	Fer	(−)	(−)	(++++)
3	Male	9	Stage III in the mediastinum, remission, relapse, died	No Fc				(−)
4	Female	13	Stage III, remission relapse, remission	Fer++	Fer	(−)	(−)	(+++)
5	Male	1	Stage IV neuroblastoma and ganglioneuroma in adrenal tissue	No Fc	Fer	No Fc	(−)	(+++)
6	Male	5	Stage IV brain (olfactory area) NB	(−)	Fer,Feb.	(−)	No Fc	(++++)
7	Male	4	Adrenal NB, mostly necrotic tissue	(−)	Fer	(−)	(−)	(+++)
8	Male	2	Stage IV, abdominal mass NB	Fer+	No Fc	(−)	(−)	(+++)
9	Male	1	Stage IV adrenal NB. Chemotherapy (+)	No Fc	Fer	(−)	(−)	(++++)
10	Male	11	Spontaneously regressed NB in mediastinum	No Fc	Fer	(−)	(−)	(+++)
11	Male	1	Undifferentiated abdominal NB	Fer+	No Fc	(−)	(−)	(+)
12	Male	67	No comments available	No Fc	Fer	(−)	(−)	(+)
13	Male	2/12	Same as above	No FC				(−)
14	Female	5	Stage IV metastatic NB to BM	**(2) Checked in bone marrow** Clusters of 4–20 Fc, mainly Feb, throughout the whole area				(+++++)
15	Male	3	Adrenal non metastatic NB	No Fc				(−)
16	Female	3	Stage IV adrenal NB metastatic to bone	No Fc				(−)
17	Male	2	Same as above	No Fc				(−)

Abbreviations: (−), tissue not present in section; BV, blood vessels; BR, normal brain tissue; Fc, F-cell; Feb, F-blasts; Fer, F-erythrocytes; Fer+, low percent of Fer; Fer++, high percent of Fer; GN, ganglioneuroma; NB, neuroblastoma.

**Table 3:** The distribution of F-cells detected immunohistochemically in retinoblastoma patients

**No**	**Gender**	**Age (years)**	**F-cells features (1) Checked in tumour tissues**			**Staining grade**
1	Male	1	In BV, 50–100% Fc, 20–50% of them Feb. Proliferation of such BV and of free Fer,Feb clusters. Congestions of Fc in haemorrhagic regions. Some Feb in mitosis, or as binucleated.			(+++)
2	Male	1	Same as above			(+++++)
3	Male	1	Same as above			(+++++)
4	Male	2	Same as above			(+++++)
5	Male	1	BV	PBV	TC	
			Fer++	(−)	Fer	(+++++)
6	Male	1/12	Fer++	(−)	Fer,Feb	(++++)
7	Female	4	Fer+	(−)	Feb	(+++++)
8	Female	1	No Fc	(−)	Feb	(+++++)
9	Male	11	(−)	Fer+	No Fc	(+++)
10	Male	2	No Fc	(−)	Feb	(+)
11	Female	5	No Fc	(−)	Feb	(++)
12–14	1 Male 2 female	1, 2, 4	No Fc			(−)
15	Female	4	**(2) Checked in bone marrow** Clusters of Fc , predominantly Feb, throughout the whole section.			(+++++)
16	Female	3	Same as above			(+++++)
17	Female	4	Same as above			(+++++)
18	Male	3	No Fc			(−)

Abbreviations: (−), tissue not present in section; BV, blood vessels; Feb, Fblasts; Fc, F-cells; Fer, F-erythrocytes; Fer+, low percent of Fer; Fer++, high percent of Fer; PBV, proliferating blood vessels; TC, tumour tissue.

**Table 4:** The distribution of F-cells immunohistochemically detected in rhadomyosarcoma

				**F-cells features (1) Checked tumour tissues**			
**No.**	**Gender**	**Age (years)**	**Tumour type**	**BV**	**PBV**	**TC**	**Staining grade**
1	Female	2	Alveolar	(−)	Fer,Feb++	Fer,Feb	(+++++)
2	Female	3	Embryonal	Fer++	(−)	No Fc	(++++)
3	Female	4	Alveolar	Fer++	(−)	Fer	(++++)
4	Male	1	Embryonal	Fer++	(−)	No Fc	(+++++)
5	Female	5	Embryonal	(−)	(−)	Fer,Feb	(++++)
6	Female	30	Embryonal	Fer+	(−)	No Fc	(++++)
7	Male	51	Embryonal	Fer+	(−)	No Fc	(++)
8	Male	8	Embryonal	(−)	(−)	Fer	(+)
9	Male	3	Embryonal. Chemt(+)	No F-cells			(−)
10	Male	13		(2) Checked in bone marrow clusters of infiltrating Fc, mainly Fer, throughout the whole section			(+++++)
11	Female	6		Clusters of infiltrating Fc, mainly Feb, some Feb in mitosis			(+++++)
12	Female	11	Mets to bone marrow	No Fc			(−)
13	Female	25	Same as above	No Fc			(−)
14	Male	2		No Fc			(−)

Abbreviations: (−), tissue not present in section; BV, blood vessels; Fc, F-cells; Feb, F-blasts; Fer, F-erythrocytes; Fer+, low percent of Fer; Fer++, high percent of Fer; PVB, proliferating blood vessels; TC, tumour tissue.

**Table 5:** The distribution of F-cells immunohistochemically detected in medulloblastoma of the cerebellum

			**F-cells features**		
**No.**	**Gender**	**Age (years)**	**BV**	**TC**	**Staining grade**
1	Female	12	Fer++	Fer	(+++)
2	Female	11	Fer+	Fer	(+++)
3	Female	48		Fer	(+++)
4	Female	35	Fer++	Feb	(+++)
5	Male	26	(−)	Fer	(+)
6	Female	5	(−)	Feb	(+)
7	Female	5	No Fc		(−)
8	Male	26	No Fc		(−)
9	Male	26	No Fc		(−)
10	Female	27	No Fc		(−)
11	Female	15	Bone marrow with no Fc		(−)

Abbreviations: (−), tissue not present in section; BV, blood vessels; Fc, F-cells; Feb, F-erythroblasts; Fer, F-erythrocytes; Fer+, low percent of Fer; Fer++, high percent of Fer.

**Table 6:** The incidence of bone marrow F-cells in leukaemia patients

**Leukaemia type**	**F-cells positive**	**F-cells negative**	**Total**
*In children 2–14 years old*			
ALL	5	1	6
AML	0	1	1
			
*In adults 31–81 years old*			
ALL	0	1	1
AML	2	1	3
CLL	4	1	5
CML	6	0	6
Hairy cell leukaemia	2	0	2
Total	19	5	24
